# Adaptive engineering of a hyperthermophilic archaeon on CO and discovering the underlying mechanism by multi-omics analysis

**DOI:** 10.1038/srep22896

**Published:** 2016-03-15

**Authors:** Seong Hyuk Lee, Min-Sik Kim, Jae-Hak Lee, Tae Wan Kim, Seung Seob Bae, Sung-Mok Lee, Hae Chang Jung, Tae-Jun Yang, Ae Ran Choi, Yong-Jun Cho, Jung-Hyun Lee, Kae Kyoung Kwon, Hyun Sook Lee, Sung Gyun Kang

**Affiliations:** 1 Korea Institute of Ocean Science and Technology, Ansan, Republic of Korea; 2 Department of Marine Biotechnology, Korea University of Science and Technology, Daejeon, Republic of Korea; 3 Korea Institute of Energy Research, Daejeon, Republic of Korea; 4 Chunlab, Inc., Seoul, Republic of Korea

## Abstract

The hyperthermophilic archaeon *Thermococcus onnurineus* NA1 can grow and produce H_2_ on carbon monoxide (CO) and its H_2_ production rates have been improved through metabolic engineering. In this study, we applied adaptive evolution to enhance H_2_ productivity. After over 150 serial transfers onto CO medium, cell density, CO consumption rate and H_2_ production rate increased. The underlying mechanism for those physiological changes could be explained by using multi-omics approaches including genomic, transcriptomic and epigenomic analyses. A putative transcriptional regulator was newly identified to regulate the expression levels of genes related to CO oxidation. Transcriptome analysis revealed significant changes in the transcript levels of genes belonging to the categories of transcription, translation and energy metabolism. Our study presents the first genome-scale methylation pattern of hyperthermophilic archaea. Adaptive evolution led to highly enhanced H_2_ productivity at high CO flow rates using synthesis gas produced from coal gasification.

Microorganisms have been metabolically engineered, employing integrated strategies of systems biology, synthetic biology and evolutionary engineering, to enhance their output of valuable products^1^. Generally, metabolic engineering via knowledge-based rational design has been used to enhance the output of target products; however, this approach requires *a priori* genetic or biochemical information, and the complexity of cellular physiological responses must be considered^2^. In this regard, an evolutionary engineering approach may serve as an alternative for obtaining suitable phenotypes in cases where there is a lack of prior knowledge^3^,^4^,^5^,^6^. This approach has been applied to many organisms^7^, including *Escherichia coli*, *Myxococcus xanthus*, *Pseudomonas fluorescens*, *Saccharomyces cerevisiae* and *Thermotoga maritima*^8^. However, it has never been reported for hyperthermophilic archaea.

Biohydrogen has the potential to considerably reduce costs and environmental impact because it can be produced using sunlight, minimal nutrients or organic waste effluents^9^. Microbial H_2_ production by dark fermentation can produce H_2_ at a faster rate than photosynthesis and can upcycle renewable, cheap and abundant resources^10^. *T. onnurineus* NA1 is a carboxydotrophic hydrogenogen, capable of utilizing CO and producing H_2_ in the following reaction: CO + H_2_O → H_2_ + CO_2_ (ΔG°′ = −20 kJ/mol)^11^. CO-driven H_2_ production is mediated by an enzymatic system encoded in the CODH gene cluster, composed of a carbon monoxide dehydrogenase (CODH), a hydrogenase and a Na^+^/H^+^ antiporter^12^,^13^,^14^. We have enhanced the H_2_ production rate of the strain through knowledge-based rational design, such as by overexpressing genes in the CODH gene cluster by promoter engineering or by CO regulator engineering^14^,^15^,^16^,^17^. Although the design was successful, the resulting mutant strains could not grow in a fermentor supplied with a CO flow rate above 800 ml min^−1^. Therefore, the development of a mutant with improved performance at high CO flow rates is desirable.

In the current study, we utilized evolutionary engineering to optimize *T. onnurineus* NA1 under CO conditions and investigated the underlying mechanism of optimization by employing multi-omics analysis. Genetic variations and transcriptomic and epigenomic changes were analyzed using next generation sequencing. The methylation profile of NA1 was monitored via SMRT DNA sequencing^18^. DNA methylation has been reported to be involved in the regulation of genome replication^19^. Genome-scale methylation patterns of some bacteria and archaea have been published^20^,^21^,^22^. However, genomic scale interpretation of base modifications has not yet been reported for hyperthermophiles. Kinetic analysis of H_2_ production in the adapted strain was conducted and compared with other genetically engineered strains.

## Results

### Physiological changes in *T. onnurineus* NA1 during adaptive evolution on CO

**Figure 1:**
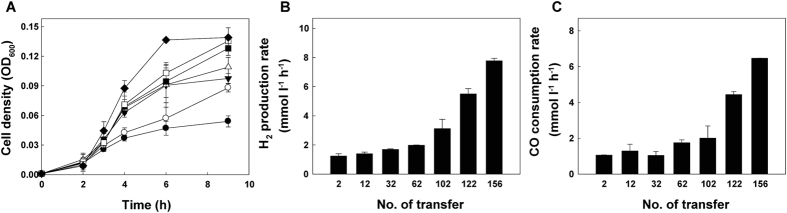
Physiological changes in *T. onnurineus* NA1 following serial transfers into fresh MM1 medium containing 100% CO. After 2 (closed circle), 12 (open circle), 32 (closed inverted triangle), 62 (open triangle), 102 (closed square), 122 (open square) and 156 (closed diamond) transfers, (**a**) cell density (expressed as optical density at 600 nm) was determined at the indicated timepoints. (**b**) H_2_ production rates and (**c**) CO consumption rates were determined during exponential phase. All experiments were conducted independently in duplicate.

We applied an evolutionary approach to enable *T. onnurineus* NA1 to adapt to 100% pure CO. *T. onnurineus* NA1 was grown to stationary phase to trigger spontaneous mutation^23^,^24^,^25^,^26^ and then transferred to fresh medium containing CO as an energy source; the strain was transferred over 150 times. Through these serial transfers, physiological changes were monitored, and gradual increases in cell density, H_2_ production rate and CO consumption rate were observed (Fig. 1). After 156 transfers, the evolved strain, designated 156 T, showed 2.8-, 6.5- and 5.9-fold higher values in maximum cell density, H_2_ production rate and CO consumption rate, respectively, than the parental strain (2 T) (Fig. 1). The correlation between the increases in CO consumption rate and H_2_ production rate seemed to reflect that H_2_ production occurred by hydrogenogenic carboxydotrophy. The enhanced cell growth was associated with the increases in CO consumption rate and H_2_ production, concomitant with energy conservation via the conversion of CO oxidation.

### Genomic analysis

The genomic DNA of 156 T was isolated and sequenced using Illumina Hi-seq2000 and SMRT DNA sequencing. In comparison with the sequence of the parental strain, several mutations, including base substitutions, deletions and insertions, were identified and verified by Sanger sequencing (Table 1, Supplementary Fig. 1). The mutation list includes base substitutions, leading to amino acid changes, in aromatic amino acid permease (TON_0820), transcriptional regulator (TON_1525) and membrane protein (TON_1544) and single- or multiple-base deletions/insertions in aminotransferase (TON_0982), hypothetical protein (TON_1548), cation transporter (TON_1664), metalloprotease (12 bp inserted) (TON_1694), and alcohol dehydrogenase (338 bp deleted) (TON_0544), as well as in the intergenic region (51 bp deleted) between sodium/phosphate symporter (TON_1475) and pyruvate/ketoisovalerate ferredoxin oxidoreductase (TON_1476). A long deletion (5786 bp deleted) between cytosolic NiFe-hydrogenase subunit gamma (TON_0536) and 4Fe-4 S binding protein (TON_0541) also occurred (Supplementary Fig. 2).

### Genome-wide transcriptional analysis

**Figure 2:**
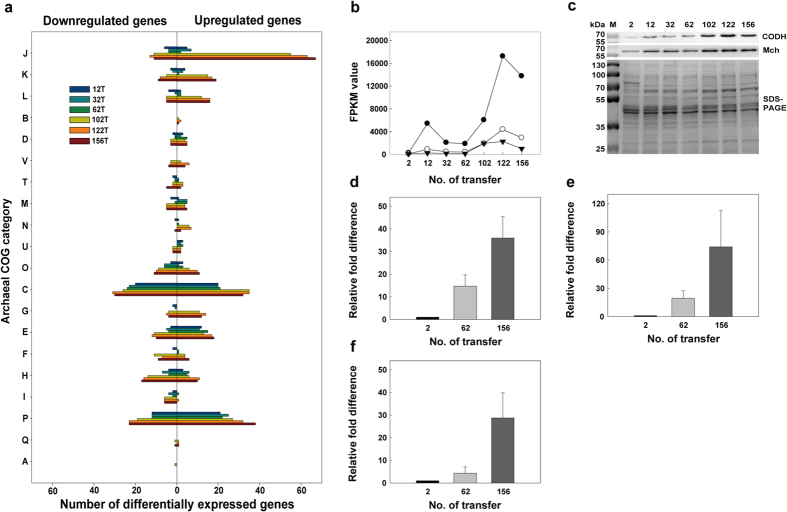
Archaeal COG classification of differentially expressed genes and changes in the expression levels of genes in the CODH gene cluster during adaptation. (**a**) The following COG categories were included: J, Translation, ribosomal structure and biogenesis; K, Transcription; L, Replication, recombination and repair; B, Chromatin structure and dynamics; D, Cell cycle control, cell division and chromosome partitioning; V, Defense mechanisms; T, Signal transduction mechanisms; M, Cell wall/membrane/envelope biogenesis; N, Cell motility; U, Intracellular trafficking, secretion and vesicular transport; O, Posttranslational modification, protein turnover and chaperones; C, Energy production and conversion; G, Carbohydrate transport and metabolism; E, Amino acid transport and metabolism; F, Nucleotide transport and metabolism; H, Coenzyme transport and metabolism; I, Lipid transport and metabolism; P, Inorganic ion transport and metabolism; Q, Secondary metabolite biosynthesis, transport and catabolism; A, RNA processing and modification. The numbers of upregulated genes and downregulated genes are indicated as bars in the right and left panels of the histogram, respectively. (**b**) RNA-seq analysis of genes encoding CODH (TON_1018) (closed circle), Mch (TON_1023) (open circle) and Mnh (TON_1031) (closed inverted triangle). (**c**) Western blot analysis of TON_1018 (67.7 kDa) and TON_1023 (61.7 kDa). RT-qPCR analysis of mRNA abundance of TON_1018 (**d**), TON_1023 (**e**) and TON_1031 (**f**). Error bars indicate standard deviations from duplicate experiments. FPKM, Fragments per kilobase per million mapped reads; M, molecular mass marker.

Transcriptional changes were monitored by RNA-seq using RNA samples from the strains produced after 2, 12, 32, 62, 102, 122, and 156 transfers. The differentially expressed genes in each transferred strain were selected using the DESeq and edgeR packages after being compared against the transcript levels in the 2 T strain. As the transfer process continued, the number of differentially expressed genes (DEGs) gradually increased. Approximately 200 and 500 DEGs were found in the early stages of transfer (12 T, 32 T, 62 T) and late stages of transfer (102 T, 122 T, 156 T), respectively. To interpret the biological and functional meanings of these transcriptional changes, a total of 819 DEGs were categorized according to archaeal clusters of orthologous genes (arCOGs)^27^ (Fig. 2a).

As the transfer process progressed, we observed remarkable changes in the number of upregulated DEGs in the COG J (translation, ribosomal structure and biogenesis), COG K (transcription) and COG L (replication, recombination and repair) groupings, together belonging to the category ‘information storage and processing’ (Fig. 2a and Supplementary Data 1). The gradual increase in DEG expression concomitant with the adaptation process seems reasonable because the overexpression of genes encoding ribosomal proteins, RNA polymerase subunits, DNA polymerase II subunits, translation/transcription initiation or elongation factors and DNA topoisomerase subunits supports the observed faster growth and increased growth yield.

Gene clusters encoding seven hydrogenases, SulfI, SulfII, Mbh, Mfh2, Mch and Frh, and one oxidoreductase, Mbx, homologous to hydrogenase^28^, were included in the DEG list; they fell under the COG C (energy production and conservation) grouping. The expression levels of the ATP synthase genes (TON_1749–1753) gradually increased during the adaptation. In particular, genes encoding CODH-Mch subunits (TON_1017–1024) that are essential for hydrogenogenic carboxydotrophy were significantly upregulated at both the transcriptional and translational level as the transfer process progressed (Fig. 2, Supplementary Fig. 3a). The CODH-Mch complex has been proposed to oxidize CO, thereby conserving energy by generating an electrochemical gradient^14^. The electrochemical gradient is eventually coupled to ATP generation by ATP synthase; therefore, the overexpression of CODH-Mch and ATP synthase could allow cells to generate more ATP. The expression of other gene clusters did not persistently increase during the adaption. The Mbh genes (TON_1583–1595) were strongly upregulated during the early stages of adaptation, but their transcript levels during the late stages of adaption were similar to those in the 2 T strain. The Mbx genes (TON_0486–0498) were only upregulated at late stages. The Mbh and Mbx might be necessary to dispose of or recycle reduced ferredoxin generated from amino acid degradation. Conversely, the genes encoding SulfI (TON_0534–0537), SulfII (TON_0052), Mfh2 (TON_1564–1580) and Frh (TON_1560–1561) showed significant downregulation throughout the entire adaptation period (Supplementary Fig. 3b). Mfh2 has been reported to participate in energy conservation using electrons generated from formate oxidation, and two soluble hydrogenases, SulfI and SulfII, have been proposed to uptake H_2_ to enhance reducing power^29^,^30^. During the adaptation period, the cells seemed likely to upregulate the genes necessary for growth on CO and to downregulate the genes not needed for this growth.

The upregulated genes in the COG P (inorganic ion transport and metabolism) grouping included two gene clusters encoding Na^+^/H^+^ antiporters (TON_1021–1031, TON_1582–1589) in proximity to the Mch and Mbh hydrogenases (Supplementary Fig. 3a). These antiporters have been proposed to form a complex with hydrogenase subunits that exchanges proton gradients into sodium gradients; these gradients are eventually coupled with ATP generation by a sodium-specific ATP synthase^31^. The transcript patterns of these antiporter genes were correlated with those of their cognate hydrogenase gene clusters. Genes encoding iron transporters (TON_0255–0256, 0984, 1873) and a peptide transporter (TON_1765–1768) exhibited increased transcript levels, likely to enhance cellular uptake of iron and peptides to facilitate cell growth. The gene encoding the cell envelope S-layer protein (TON_0413) was also upregulated together with genes (TON_1837, 1842–1843, 1848, 1851) involved in the synthesis of dTDP-_L_-rhamnose, a substrate used for the glycosylation of S-layer protein^32^ (Supplementary Fig. 4).

### Genome-wide epigenomic analysis

**Figure 3:**
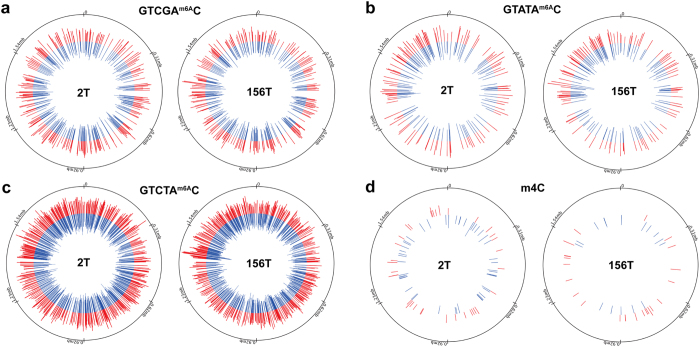
Comparison of the methylation profiles of adenine and cytosine residues between the 2T and 156T strains. Only positions with *p*-values lower than 10^−4^ (score ≤ 40) and larger than 25 (coverage > 25) were selected as modified bases. The locations of methylated adenines within GTCGA^m6A^C (**a**), GTATA^m6A^C (**b**) and GTCTA^m6A^C (**c**) and of methylated cytosine, m4C (**d**), are displayed in the 2 T (left panel) and 156 T genomes (right panel) as bars. The length of each bar represents the level of confidence in the methylation (−10*log[P-value]). Methylations in the + and − strands are indicated by red and blue bars, respectively. In the outermost circles, the locations dividing the genome into 6 equal parts are indicated as ticks.

Adenine and cytosine methylation patterns in the genomes of the 2 T and 156 T strains were analyzed by SMRT DNA sequencing^18^ (Fig. 3). In both strands of the 2 T and 156 T genomes, 2190 and 2105 sites of N^6^-methyl adenine (m6A) were respectively detected. Adenine methylation remained virtually the same, and more than 95% of the methylated sites (2017 out of 2190) in the 2 T strain were also detected in the 156 T strain, while the number of hemi-methylated sites increased as the adaptation process progressed. After analyzing the sequences surrounding the modified sites by MEME (Supplementary Table 1), more than 95% of the adenine methylation sequences were identified to contain a (GT(C/A)(G/T)AC) motif, which corresponds to the *Acc*I restriction enzyme site. The *Acc*I restriction enzyme (TON_1382) and its cognate methyltransferase (TON_1383) are encoded in the *T. onnurineus* NA1 genome.

Conversely, the cytosine residue methylation pattern significantly changed. In the 2 T and 156 T strains, 83 and 40 cytosine residues methylated at the N4 site of the cytosine base (N^4^-methylcytosine, m4C) were respectively identified (Supplementary Data 2). Among the 40 methylated cytosines in the 156 T strain, 36 of the methylated cytosines were not modified in the 2 T strain. The target sequence for cytosine methylation was not as clear as that for adenine methylation, but G^m4C^C was likely to be preferentially methylated over other possible sequences, such as T^m4C^C, A^m4C^C and C^m4C^C. More than 50% of the cytosine methylation sites possessed G^m4C^C sequences in both the 2 T (53 sites) and 156 T (24 sites) strains.

In the promoter region of the Mfh2 hydrogenase gene cluster, two distinct methylation sites were identified only in the 156 T strain. The methylated cytosine residues were adjacent to the DNA-binding motif (GTTNNNAAC) for a putative sulfur response regulator, SurR (Supplementary Fig. 6a), that has been reported in *Pyrococcus furiosus*^33^. One methylated adenine residue was detected inside an inverted repeat. Although it was not experimentally proven, the methylation of this residue might affect the binding of SurR and an unknown transcriptional regulator to the Mfh2 promoter, resulting in the downregulation of the gene cluster. After a few transfers, the transcript levels of the genes in the Mfh2 gene cluster were drastically downregulated (Supplementary Fig. 6b–d).

### Effects of mutations on adaptive physiological changes

**Figure 4:**
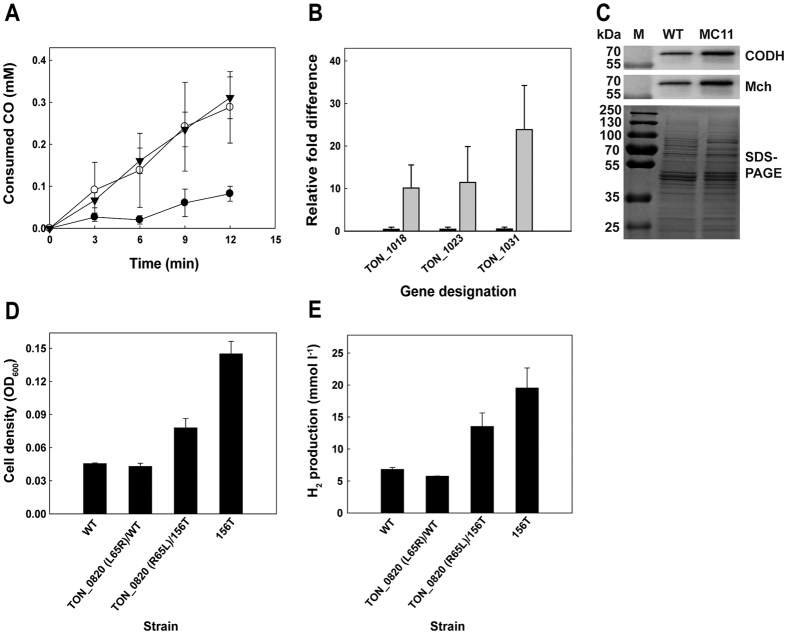
The effects of the T55I mutation in TON_1525 and the L65R mutation in TON_0820. (**a**) CO consumption was analyzed by myoglobin assay for wild-type (WT) (closed circle), MC11 (open circle) and 156T (closed inverted triangle) strains. (**b**) RT-qPCR analysis of TON_1018, TON_1023 and TON_1031 in WT (black bar) and MC11 (gray bar) strains. (**c**) Western blot analysis of TON_1018 (67.7 kDa) and TON_1023 (61.7 kDa) in WT and MC11 strains. (**d**) Comparison of cell density (expressed as optical density at 600 nm) and (**e**) H_2_ production of the TON_0820 mutants in comparison with the WT and 156T strains. Error bars indicate the standard deviations of independent triplicate (**a**,**b**) or duplicate (**d**,**e**) experiments.

To determine the mutations critical to physiological change, we tracked the distributions of seven mutations within the coding regions of selected transferred strains. The timepoint when each mutation initiated was scattered from 12th transfer to 156th transfer, and the distribution of each mutation increased as the number of transfers increased, except for the mutation in the aminotransferase gene (TON_0982) (Supplementary Fig. 7). Considering the pattern of changes in H_2_ production rate and CO consumption rate, that is, small increases during early stages and then big increases after 102nd transfer, the distributions of mutations of the genes encoding putative transcriptional regulator (TON_1525) and aromatic amino acid permease (TON_0820) among total population seemed well matched with those physiological changes. Therefore, the TON_1525 and TON_0820 mutations found in strain 156 T were introduced into the parental strain. The resulting mutant, TON_1525 (T55I)/WT (designated as MC11), showed a higher CO consumption rate than the wild-type strain; this rate was comparable to that of the 156 T strain (Fig. 4a). The increased CO consumption in the MC11 strain was likely related to the increased transcript levels of the genes in the CODH gene cluster. The transcript levels of the TON_1018, TON_1023, and TON_1031 genes were respectively 20.6-, 25.4-, and 47.7-fold higher than the levels in the parental strain (Fig. 4b). Consistent with the transcriptional changes, CODH (TON_1018) and Mch (TON_1023) protein expression significantly increased in the MC11 mutant (Fig. 4c). These results indicate that the mutation in the TON_1525 gene played a crucial role in causing physiological change by enhancing the transcript levels of the CODH gene cluster.

To investigate whether the TON_1525 protein could act like a DNA binding protein on the CODH promoter and whether mutation of TON_1525 affected its binding affinity, wild-type and T55I recombinant TON_1525 proteins were expressed and purified from *E. coli*. The unmodified recombinant TON_1525 protein specifically bound to the CODH promoter, while the binding affinity of the T55I mutant protein was less than that of the wild-type protein, as shown by competition electrophoretic mobility shift assay (EMSA) (Supplementary Fig. 5). At present, TON_1525 can be predicted to function as a transcriptional repressor on the CODH promoter, as mutation of TON_1525 decreased its binding affinity toward the regulator of the promoter and elevated the transcript level of the downstream gene cluster.

The effect of the mutation of TON_0820 was not clearly shown when we assessed a TON_0820 (L65R)/WT mutant. However, when we reverted the mutation (L65R) in the 156 T strain back to the wild-type sequence (R65L), creating TON_0820 (R65L)/156 T, significant decreases in cell density and H_2_ production were observed (Fig. 4d,e). This result suggests that the mutation of TON_0820 is another critical factor underlying the observed physiological changes that occurred during the adaptive evolution process, probably in combination with other factors.

### Kinetic measurements of CO consumption and H_2_ production

Based on the phenotype developed by the 156 T strain following serial transfers under CO conditions, the H_2_ production potential of the strain was tested in a bioreactor where 100% CO was continuously fed at a high flow rate. The 156 T strain showed a 7- and 5.2-fold higher maximum biomass yield and H_2_ production rate, respectively, when grown under CO at a flow rate of 400 ml min^−1^ than those of the wild-type strain (Supplementary Fig. 8a,b). The growth yield based on the optical cell density measured at 600 nm was 5.5, which is very exceptional for a hyperthermophilic archaeon. The 156 T strain also showed growth and produced H_2_ at flow rates of 800 ml min^−1^ and above, whereas the rationally designed mutants could not grow, indicating that adaptation rendered the 156 T strain less sensitive to high CO levels (Supplementary Fig. 8c,d). It is noteworthy that the 156 T strain displayed the highest H_2_ production rate and specific H_2_ production rate among the previously reported mutants (Table 2).

H_2_ production by the 156 T strain was investigated using synthetic gas (syngas) derived from coal gasification. The H_2_ production rate and specific H_2_ production rate linearly correlated with the flow rate of syngas (Supplementary Table 2). Considering the composition of the syngas, which is comprised of a low CO level and contaminated with CH_4_, N_2_, CO_2_ and H_2_, the H_2_ production rates, calculated by subtracting the H_2_ content of the syngas, were considerable in comparison with the values obtained under conditions with 100% CO.

Taken together, these results illustrate that adaptive evolution is a powerful method of developing a carboxydotrophic hydrogenogen with enhanced H_2_ production from CO.

## Discussion

In the current study, we performed evolutionary engineering of a hyperthermophilic archaeon, *T. onnurineus* NA1, known as a carboxydotrophic hydrogenogen^14^ or formate-oxidizing archaeon^34^, to enhance H_2_ production from CO. Over 150 serial transfers of cell cultures led *T. onnurineus* NA1 to adapt to possess better utilization of CO and higher production of H_2_, associated with increased cell density.

Genomic analysis revealed a total of ten mutations over 2000 generations. The mutation rates of *T. onnurineus* NA1 were estimated to be approximately 0.00097 ± 0.00052 per replication^35^ and 0.0018 per genome per replication^36^. Considering that the mean mutation rates of *E. coli* and *S. cerevisiae* are 1.06 and 1.39 per 100 generations, respectively^7^, the mutation rate of *T. onnurineus* NA1 was quite low, reflecting that thermophilic microbes possess mechanisms to ensure genetic fidelity, a crucial component of the ability to sustain life under extreme environments^35^,^36^. It is noteworthy that a long deletion, encompassing approximately 5.8 kb of coding region, occurred very early after the 12th transfer.

Our study unveiled two mutations in the TON_1525 and TON_0820 genes that played pivotal roles in the adaptive evolution of *T. onnurineus* NA1 on CO. TON_1525 belongs to a family of Tfx DNA-binding proteins that has not been well studied. The Tfx protein that has been identified in *Methanobacterium thermoautotrophicum* consists of two domains, a basic DNA-binding domain with a helix-turn-helix motif and an acidic domain possibly used for transcriptional activation^37^. Multiple sequence alignment revealed that TON_1525 showed only 22.9% sequence identity to Tfx from *M. thermoautotrophicum*. A BLAST search revealed that TON_1525 showed sequence identities of over 30% toward a set of genes annotated as a transcriptional regulator or, a DNA-binding protein of *Thermococcales* species and methanogens. Based on EMSA data, TON_1525 seems likely to play a role as a transcriptional repressor on the CODH promoter. Previously, a CO-responsive regulator, CorQR, was reported to activate the expression of the CODH gene cluster^15^. We did not detect any mutations in CorQR during the adaptation process. Another crucial mutation at TON_0820 seemed contributed to enhancing final cell density (Fig. 4d,e). L65R alteration of wild-type strain did not show much effect, however, R65L mutation in 156 T significantly decreased final cell density. The TON_0820 mutation might contribute to enhancing cell density probably by supplying more aromatic amino acids. The genome of *T. onnurineus* NA1 does not encode biosynthetic pathways for aromatic amino acids such as Thr, Phe and Trp^12^. These results indicated that two mutations contribute to enhance CO consumption and fitness, however, we need consider to effect of other mutations and further experiments are required to understand overall mechanism under genomic variations on CO-containing medium. Although the distribution pattern is not so well match with physiological changes, TON_0982 or TON_1694 encoding amino transferase or a membrane associated-protease, respectively can affect cell growth by modulating the rate of amino acid/protein hydrolysis. The functionality of TON_1544 and TON_1548 could not be predicted or understood at this moment and the role of TON_1664 encoding cation transporter in archaea is unclear yet and awaits further analysis.

Epigenomic analysis revealed cytosine methylation at N4 sites in *T. onnurineus* NA1. N^4^-methylcytosine (m4C) has been discovered in both thermophilic and mesophilic bacteria^38^,^39^. Considering the high incidence of deamination at high temperatures^40^, the m4C modification may help avoid high mutation rates in organisms living at high temperatures. As deamination of m5C residues increases at high temperatures, thermophiles are unlikely to possess m5C residues in their genomes^38^. Aside from the existence of several other genes annotated as restriction enzymes and putative methyltransferases in the genome, no other consensus sequences, except the sequence encoding the *Acc*I restriction site, were discovered in proximity to methylated adenine sites. Nonetheless, it is noteworthy that the methylation profile produced in this study relied on the detection ability of SMRT DNA sequencing technology, and we cannot completely exclude the possibility that other types of methylation could exist in *T. onnurineus* NA1.

In conclusion, we performed evolutionary engineering of a hyperthermophilic archaeon using CO and demonstrated that this approach was very effective in enhancing H_2_ productivity. The underlying mechanism was unique in that a novel transcriptional regulator was identified as a crucial adaptation factor, in addition to the potential involvement of distinctive epigenomic changes. The evolved strain was superior to previously reported strains engineered by rational design. In addition to by-product gases from the steel-making process^41^, syngas from a coal gasifier was shown to be an alternative source of CO for H_2_ production by *T. onnurineus* NA1.

## Methods

### Strains, media and culture conditions

The *T. onnurineus* NA1 (KCTC10859) strain was routinely cultured in yeast extract-peptone-sulfur (YPS) medium as previously reported^42^. The modified medium 1 (MM1)^34^,^43^ was prepared with 1 g liter^−1^ yeast extract, 35 g liter^−1^ NaCl, 0.7 g liter^−1^ KCl, 3.9 g liter^−1^ MgSO_4_, 0.4 g liter^−1^ CaCl_2_·2 H_2_O, 0.3 g liter^−1^ NH_4_Cl, 0.15 g liter^−1^ Na_2_HPO_4_, 0.03 g liter^−1^ Na_2_SiO_3_, 0.5 g liter^−1^ NaHCO_3_, 0.5 g liter^−1^ cysteine-HCl, and 0.001 g liter^−1^ resazurin. One milliliter liter^−1^ of Holden’s trace element/Fe-EDTA solution^44^ and 1 ml liter^−1^ of Balch’s vitamin solution^45^ were added as a supplement to the medium. After autoclaving, the medium was kept in an anaerobic chamber (Coy Laboratory Products, Grass Lake, MI) filled with an anoxic gas mixture (N_2_:H_2_:CO_2_, 90:5:5) to equilibrate, and the final pH of the medium was adjusted to 6.5 with 2 N HCl. For the cultures in serum bottles, the media were reduced with 0.005% Na_2_S·9 H_2_O and the headspaces were filled with 100% CO (MM1-CO). The serum bottles were sealed with bromobutyl rubber stoppers and aluminum crimp caps. For the adaptive evolution, the parental strain was cultured on MM1-CO medium at 80 °C for 20 h and then a 2% (v/v) cells were inoculated into a fresh medium using a sterile syringe. At every tenth transfer, cell cultures were stoked at 4 °C for later use. For batch culture, the strain was cultured in a 3 L bioreactor (Fermentec, Cheongwon, Korea), whose working volume was 2 L at 300 rpm agitation speed. The batch culture was maintained at pH 6.1–6.2 using 0.2 M NaOH in 3.5% NaCl. Using a mass flow controller, 100% CO was supplied at feeding rates of 400, 800 or 1000 ml min^−1^ (MKPrecision, Seoul, Korea). Coal-gasified syngas (30.9% CO, 20.4% H_2_, 6.95% CO_2_, 1.42% CH_4_, and 32.1% N_2_) was provided by the Institute for Advanced Engineering in Korea and supplied at feeding rates of 160, 320, 480 or 640 ml min^−1^. The cultures were maintained under anaerobic conditions by preparing the media in an anaerobic chamber (Coy Laboratory Products, Grass Lake, USA) filled with an anoxic gas mixture (N_2_:H_2_:CO_2_, 90:5:5), and the bioreactor was sparged with pure argon gas (99.999%) through a microsparger.

### Construction of mutants

Mutants of the TON_1525 and TON_0820 genes were made by applying a gene recombination system. Briefly, we designed primer sets for base-pair substitutions and mutated the TON_1525 and TON_0820 genes by site-directed mutagenesis. Each mutated gene and its flanking regions were ligated by one-step sequence- and ligation-independent cloning (SLIC)^46^, and subsequent mutants were generated through homologous recombination using an unmarked in-frame deletion^15^ method and a modified gene disruption system that has been previously used for *Thermococcus kodakarensis* KOD1^47^. The mutations were verified by PCR using the primers listed in Supplementary Table 3 for Sanger sequencing.

### Preparation of cell suspensions and myoglobin assay

To analyze CO consumption rate, a myoglobin assay was conducted by modifying a previously reported protocol^48^,^49^. To prepare cell suspensions, cells were cultured on MM1-CO medium and then harvested by centrifugation at 7500 × *g* for 20 min at room temperature under anaerobic conditions. Cell pellets were washed twice and resuspended in MM1 medium. Final cell density (expressed as optical density at 600 nm) was measured with a BioPhotometer plus UV-Vis spectrophotometer (Eppendorf, Hamburg, Germany) and adjusted to 0.3. Aliquots of 100 μl of the cell suspension were mixed with 0.9 ml of CO-saturated MM1 medium and incubated at 80 °C. During the reaction, 10 μl of the reaction solution was collected every 3 mins and immediately added to a quartz cuvette containing a myoglobin reaction mixture that included 56 μM myoglobin (Sigma-Aldrich, St. Louis, UAS) from equine heart dissolved in 10 mM sodium thionite in 0.1 M potassium phosphate buffer (pH 7). After a 3 min incubation, the absorbance at 423 nm was measured to analyze consumed CO.

### Purification of the proteins encoded by TON_1525(WT) and TON_1525(T55I)

TON_1525 and TON_1525(T55I) plasmids were transformed into an *E. coli* BL21 Rosetta (DE3) pLysS strain. Gene expression was induced by IPTG at the mid-exponential growth phase, followed by a 3 h incubation at 37 °C. The cells were harvested by centrifugation (6,000 × *g* at 4 °C for 20 min) and resuspended in 50 mM Tris-HCl buffer (pH 8.0) containing 0.1 M KCl and 10% (v/v) glycerol. The cells were disrupted by sonication and then centrifuged at 15,000 × *g* at 4 °C for 30 min. The resulting supernatant was applied to a column loaded with TALON metal affinity resin (BD Clontech, Mountain View, CA) and washed with 10 mM imidazole in 50 mM Tris-HCl buffer (pH 8.0) containing 0.1 M KCl and 10% (v/v) glycerol. The enzyme was eluted with the same buffer but with the addition of 300 mM imidazole. Protein concentration was determined by Bradford assay, and protein purity was evaluated by sodium dodecyl sulfate-polyacrylamide gel electrophoresis (SDS-PAGE).

### Electrophoretic mobility shift assay (EMSA)

DNA probes for EMSA were produced from *T. onnurineus* NA1 genomic DNA. A 150-bp probe corresponding to the CODH promoter was amplified using the primer pair labeled_FAM_1017_150_F and 1017_150_R. For competition analysis, a specific 150-bp cold competitor probe was derived from the same CODH promoter region as above via amplification using the primer pair unlabeled_FAM_1017_150_F and 1017_150_R. Poly(deoxyinosinic-deoxycytidylic acid) (Sigma-Aldrich, St. Louis, USA) and pUC18 plasmid DNA (New England BioLabs, Ipswich, MA) digested with *Hpa* II were used as nonspecific competitors in 100× molar excess. The PCR products were purified using a Qiagen PCR purification kit (Qiagen, GmbH, Hilden, Germany). EMSA reactions including 100 nM DNA with varying amounts of protein were set up in 20 μl of EMSA buffer (20 mM Tris-HCl, 200 mM KCl, 1 mM EDTA, 5% glycerol, pH 7.5). After reaction at 80 °C for 10 min, the samples were immediately loaded onto a non-denaturing 5% polyacrylamide gel. The resulting gel was stained with ethidium bromide (EtBr) and analyzed to visualize the presence of proteins in the shifted bands. All of the primers that were used are listed in Supplementary Table 3.

### Reverse transcription-quantitative PCR (RT-qPCR) and western blotting

To eliminate genomic DNA from total RNA preparations, 8 μg of RNA was incubated with 8 units of RNase-free DNase I (Thermo Scientific Fermentas, St. Leon-Rot, Germany) at 37 °C for 30 min and purified via chloroform extraction and ethanol precipitation. RNA was quantified with a NanoDrop 2000 UV-Vis spectrophotometer (Thermo Scientific, West Palm Beach, USA), and cDNA was created from 1 μg of RNA by incubation with 40 units of Moloney murine leukemia virus reverse transcriptase (Thermo Scientific Fermentas, St. Leon-Rot, Germany), 5 μM random hexamers, and 1 mM deoxynucleoside triphosphate (dNTP) at 37 °C for 1 h in reverse transcription buffer. The reaction products were serially diluted to find the adequate concentration for real-time PCR analysis, and the samples were amplified with SYBR green real-time PCR master mix (Toyobo, Osaka, Japan). Amplified signals were detected using the StepOnePlus system (Applied Biosystems, Foster City, USA), and all primers that were used are listed in Supplementary Table 3. The relative amount of each gene was calculated based on cycle threshold (CT) values using a relative standard curve after normalization against the corresponding quantity of 16 S rRNA (TON_1979). Western blots were prepared and analyzed using a chemiluminescent dye from an Immun-Star horseradish peroxidase (HRP) chemiluminescent kit (Bio-Rad, Hercules, USA). Polyclonal antibodies were produced by immunizing rabbits with recombinant TON_1018 or TON_1023 proteins according to established procedures for producing custom rabbit antibodies (Young In Frontier Co., Seoul, Korea) and purified through His-Bind resin (Novagen, Madison, USA).

### Genome sequencing

For genome re-sequencing, we extracted genomic DNA from cultures of the 156 T strain without single-colony isolation. Genome sequencing was performed using Illumina HiSeq-2000 (Macrogen, Seoul, Korea) and PacBio Single Molecule Real-Time (SMRT) sequencing (Pacific Biosciences, Menlo Park, USA)^50^. HiSeq-2000 sequencing was performed using barcoded adaptors for multiplexed paired-end 101 bp sequencing and provided 100× coverage of the 156 T genome. The resulting genomic sequence data were processed using CASAVA-1.8.2 and BWA v0.5.9-r16 software (Illumina, San Diego, USA) for alignment. PCR duplicates were removed by Picard v1.79, and variants were detected using SAMtools v0.1.18. PacBio SMRT sequencing of a 10-kb insert library provided approximately 100× coverage. Assembly and consensus polishing were performed using SMRTpipe HGAP and SMRTpipe Quiver, respectively. All mutations were verified by PCR and Sanger sequencing, and all primers are listed in Supplementary Table 3.

### RNA extraction and RNA sequencing

RNA was prepared from exponentially growing cells that were transferred 2, 12, 32, 62, 102, 122 or 156 times into CO-containing media. RNA extraction was performed using TRIzol reagent (Invitrogen, Carlsbad, USA) according to the manufacturer’s instructions with some modifications, and the quantity and quality of the total RNA were evaluated using RNA electropherograms (Agilent 2100 Bioanalyzer, Palo Alto, USA) and RNA integrity numbers (RINs)^51^. A total of 10 μg of total RNA over an RIN value of 8.0 from each sample was used as a starting material and treated with a Ribo-Zero rRNA Removal Kit for Bacteria (Epicentre, Madison, USA). The resulting mRNA samples were processed into sequencing libraries using a TruSeq Stranded Total RNA Sample Prep Kit (Illumina, San Diego, USA) following the manufacturer’s protocol. Sequencing was performed using a HiSeq 2500 (Illumina, San Diego, USA) to generate directional, paired-end 100 base pair reads. Quality-filtered reads were mapped to a National Center for Biotechnology Information (NCBI) reference genome sequence (BioProject ID PRJNA59043) using CLC Genomics Workbench 6.5 (CLC bio, Aarhus, Denmark). The raw RNA-seq data have been deposited in the NCBI Gene Expression Omnibus (GEO, http://www.ncbi.nlm.nih.gov/geo/) under the accession code GSE73031.

### Identification of differentially expressed genes (DEGs)

To evaluate DEGs, both the DESeq and edgeR packages were used^52^,^53^. Replicate experiments were performed only for the initial culture, which was cultured only 2 times. Fold changes in gene expression in a selected series of adapted culture samples were calculated based on comparisons to the initial culture sample. A false discovery rate (FDR) of less than 0.001 and a fold change of either more than 2 or less than 0.5 were used as the criteria for selection, and only genes that passed the thresholds from both algorithms were identified as DEGs. To interpret the functions of the identified DEGs, 819 genes were classified according to the archaeal cluster of orthologous genes (arCOGs)^27^. To analyze changes in transcript patterns of pathway-related genes, the Kyoto Encyclopedia of Genes and Genomes (KEGG) database (http://www.genome.jp/kegg/) was also used.

### Methylation site analysis

Using a Covaris G-tube (Covaris, Inc., Woburn, MA, USA), we generated 20-kb DNA fragments by shearing genomic DNA and removed small fragments using an AMPureXP bead purification system (Beckman Coulter Inc, Brae, CA, USA) according to the manufacturer’s protocol. A total of 5 μg of each sample was used as input for library preparation. A SMRTbell library was constructed using a SMRTbell™ Template Prep Kit 1.0 (Pacific Biosciences, Menlo Park, USA). Using the BluePippin Size selection system (Sage Science, Inc., Beverly, MA, USA), we removed the small fragments to create a large-insert library. After a sequencing primer annealed to the SMRTbell template, the SMRTbell libraries were bound to polymerases using a DNA/Polymerase Binding kit P4 (Pacific Biosciences, Menlo Park, USA). Following the polymerase binding reaction, a MagBead Kit (Pacific Biosciences, Menlo Park, USA) was used to bind the library complex to MagBeads before sequencing. MagBead-bound complexes provide more reads per SMRT Cell. The polymerase-SMRTbell-adaptor complex was then loaded into zero-mode waveguides (ZMWs). The SMRTbell library was sequenced using 2 SMRT cells (Pacific Biosciences, Menlo Park, USA) with C2 chemistry (DNA sequencing Reagent 2.0), and 1 × 180 min movies were captured for each SMRT cell using a PacBio RS sequencing platform (Pacific Biosciences, Menlo Park, USA). To determine methylation sites, Pacific Biosciences SMRTanalysis software (version 2.1.1) was used, and the method depended on the sensitivity of the polymerase kinetics to the DNA template structure, as DNA synthesis was recorded in real time. An in silico kinetic reference and t-test based kinetic score were calculated based on interpulse duration (IPD) for detection of modified base positions.

### Bioinformatics analysis

A homology search was performed using the Basic Local Alignment Search Tool (BLAST; http://blast.ncbi.nlm.nih.gov/Blast.cgi) against a nonredundant protein database maintained by the National Center for Biotechnology Information (NCBI)^54^. Multiple sequence alignment was performed using a pairwise sequence alignment (http://www.ebi.ac.uk/Tools/psa/) tool from the European Bioinformatics Institute (EBI).

## Additional Information

**How to cite this article**: Lee, S. H. *et al.* Adaptive engineering of a hyperthermophilic archaeon on CO and discovering the underlying mechanism by multi-omics analysis. *Sci. Rep.* **6**, 22896; doi: 10.1038/srep22896 (2016).

## Supplementary Material

Supplementary Information

Supplementary Dataset 1

Supplementary Dataset 2

## Figures and Tables

**Table 1:** Mutations found in the 156 T strain.

Locus_tag	Genome position	Timeline of the mutation	Mutation type^a^	Codon change	Description
TON_0536–0541	490749–496535	after 12^th^ transfer	5786-bp deletion	frame shift	cytosolic NiFe-hydrogenase, formate transporter, formate dehydrogenase, iron-sulfur binding proteins
TON_0820	760634	after 12^th^ transfer	substitution	Leu to Arg	aromatic amino acid permease
TON_1525	1400830	after 12^th^ transfer	substitution	Thr to Ile	putative transcriptional regulator
TON_1544	1420154	after 62^nd^ transfer	substitution	Pro to Leu	membrane protein
TON_0982	906462	after 32^nd^ transfer	T insertion	frame shift	aminotransferase
TON_1548	1422235	after 102^nd^ transfer	T deletion	frame shift	hypothetical protein
TON_1664	1527982	after 156^th^ transfer	A deletion	frame shift	cation transporter
TON_1694	1559247	after 102^nd^ transfer	12-bp insertion	frame shift	membrane-associated metalloprotease
TON_0544	499427–499765	after 156^th^ transfer	338-bp deletion	frame shift	alcohol dehydrogenase
TON_1475–1476	1351559–1351609	after 62^nd^ transfer	51-bp deletion	–	intergenic region

^a^All mutations were confirmed by PCR verification and Sanger sequencing.

**Table 2:** Comparison of H_2_ production rates between wild-type and mutant strains of *T. onnurineus* NA1.

Organism	Strategy	H_2_ production rate (mmol l^−1^ h^−1^)	Specific H_2_ production rate (mmol g^−1^ h^−1^)	Reference
156T	Evolutionary engineering	220.8^c^	334.6^c^	This study
ΔCorR/*corQR*^↑^	Transcriptional regulator engineering	191.9^a^	249.6^a^	15
KS0510	Promoter engineering	155.1^b^	245.1^b^	16
MC01	Promoter engineering	123.5^b^	194.7^b^	14
wild-type	–	32.9^a^	151.3^a^	14

^a–c^100% CO was continuously fed at flow rates of 240 (MC01 and KS0510 strains), 400 (wild-type and ΔCorR/*corQR*^↑^ strains) or 800 ml min^−1^ (156T strain).
