# Supplementary material for: Adaptive engineering of a hyperthermophilic archaeon on CO and discovering the underlying mechanism by multi-omics analysis
Source: Sci Rep. 2016 Mar 15;6:22896. doi: 10.1038/srep22896 (PMC4791640; doi:10.1038/srep22896)
Supplement: Supplementary Information [file srep22896-s1.pdf]

Supplementary Information

Adaptive engineering of a hyperthermophilic archaeon on CO and discovering the underlying mechanism by multi-omics analysis

Seong Hyuk Lee[1,2,*], Min-Sik Kim[3,*], Jae-Hak Lee[1,*], Tae Wan Kim[1,2], Seung Seob Bae[1], Sung-Mok Lee[1], Hae Chang Jung[1,2], Tae-Jun Yang[1], Ae Ran Choi[1], Yong-Jun Cho[4], Jung-Hyun Lee[1,2], Kae Kyoung Kwon[1,2], Hyun Sook Lee[1,2] & Sung Gyun Kang[1,2]

[1]Korea Institute of Ocean Science and Technology, Ansan, Republic of Korea.

[2]Department of Marine Biotechnology, Korea University of Science and Technology, Daejeon, Republic of Korea.

[3]Korea Institute of Energy Research, Daejeon, Republic of Korea.

[4]Chunlab, Inc., Seoul, Republic of Korea.

**These authors contributed equally to this work.*

** To whom correspondence should be addressed : leeh522@kiost.ac.kr and sgkang@kiost.ac.kr.*

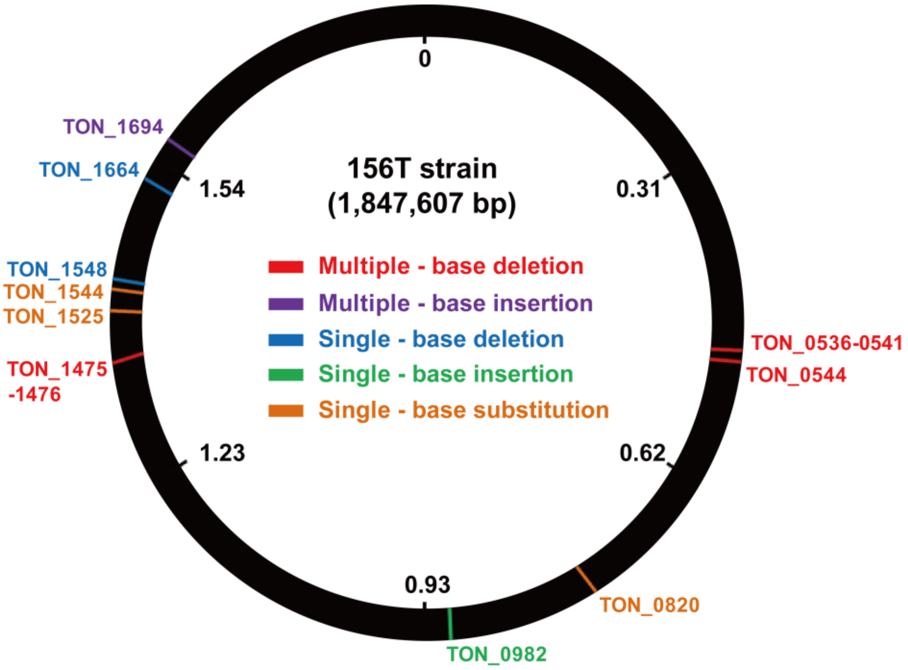

Supplementary Figure 1. Genomic mutations in the 156T strain. Mutations were analyzed using High-seq 2000 and PacBio SMRT DNA sequencers and verified by PCR and Sanger sequencing. The numbers inside and outside of the circle represent genome position (Mb) and locus tag, respectively. The mutations are summarized in Table 1.

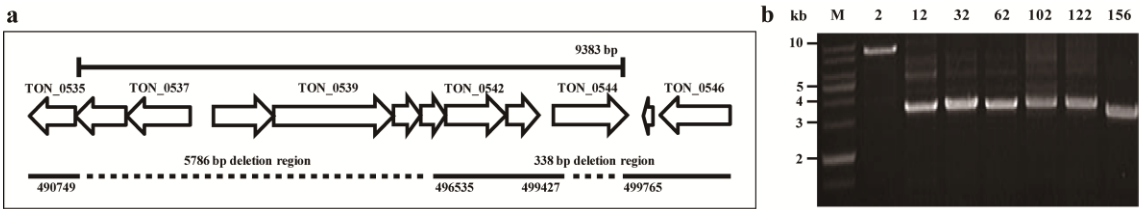

Supplementary Figure 2. Verification of the deletion of a long DNA region. (**a**) Schematic diagram of a long deletion (9383 bp) region found in the 156T strain. (**b**) Size analyses of the target sequences amplified from the genomic DNA of each transferred strain. The 5786-bp deletion spanning from 490749 to 496535 was detected in all of the transferred strains, while the 338-bp deletion spanning from 499427 to 499765 was only detected in the 156T strain.

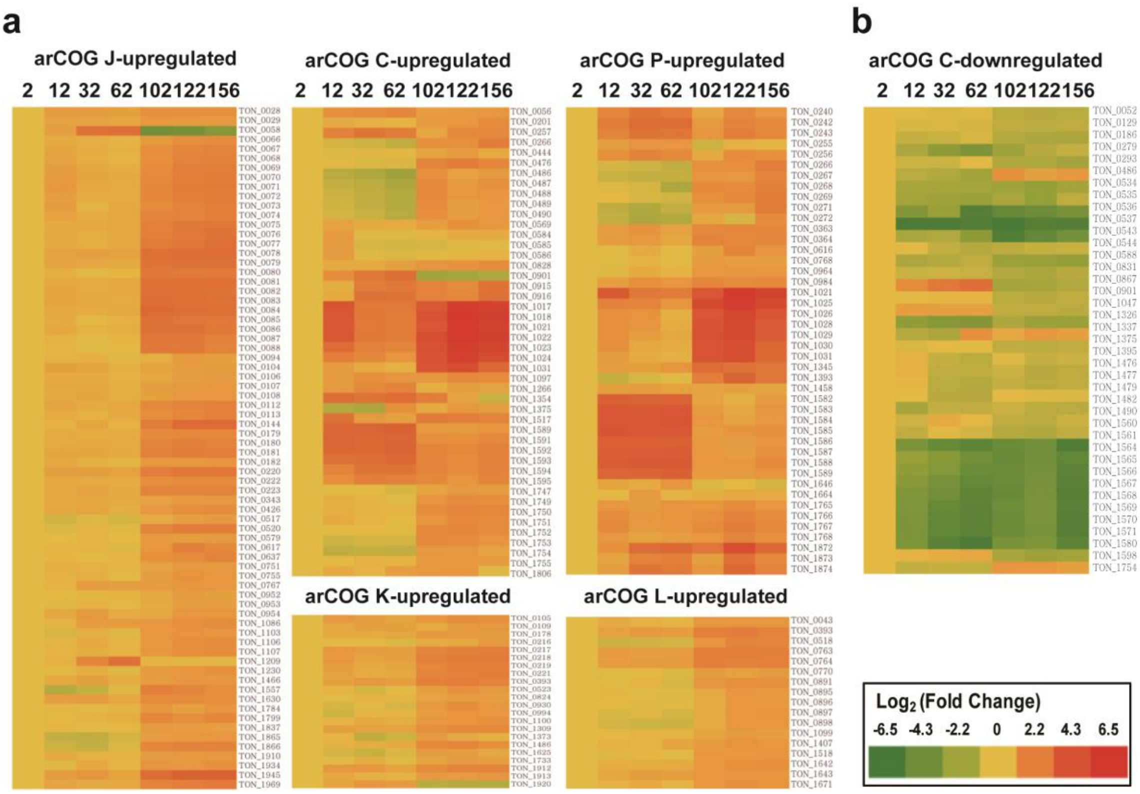

Supplementary Figure 3. Expression profiles of the genes belonging to archaeal COG J, C, P, K, L (a) and C (b). Log2 (fold change) values were extracted from DESeq data and displayed as a heatmap. The numbers at the tops of the heatmaps represent the numbers of times the cells were transferred into the MM1-CO medium. The values for TON_0538, 0540 and 0541 included in arCOG C were omitted because the fold change values could not be calculated due to a multiple-base deletion.

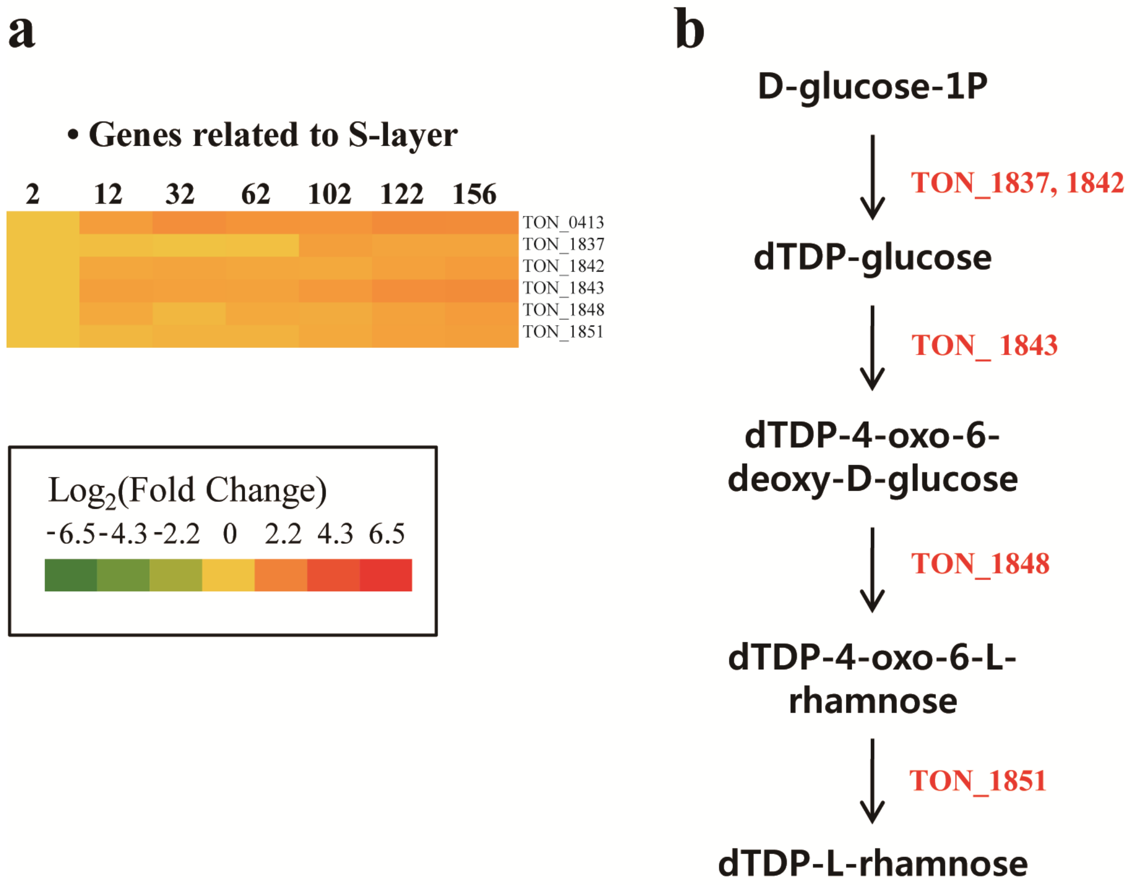

Supplementary Figure 4. Transcript patterns of the genes involved in the synthesis of an S-layer. (**a**) Changes in the transcript levels of genes related to the synthesis of an S-layer are displayed. (**b**) The pathway for the synthesis of dTDP-$_{L}$-rhamnose. The gene catalyzing each step is indicated by a locus tag.

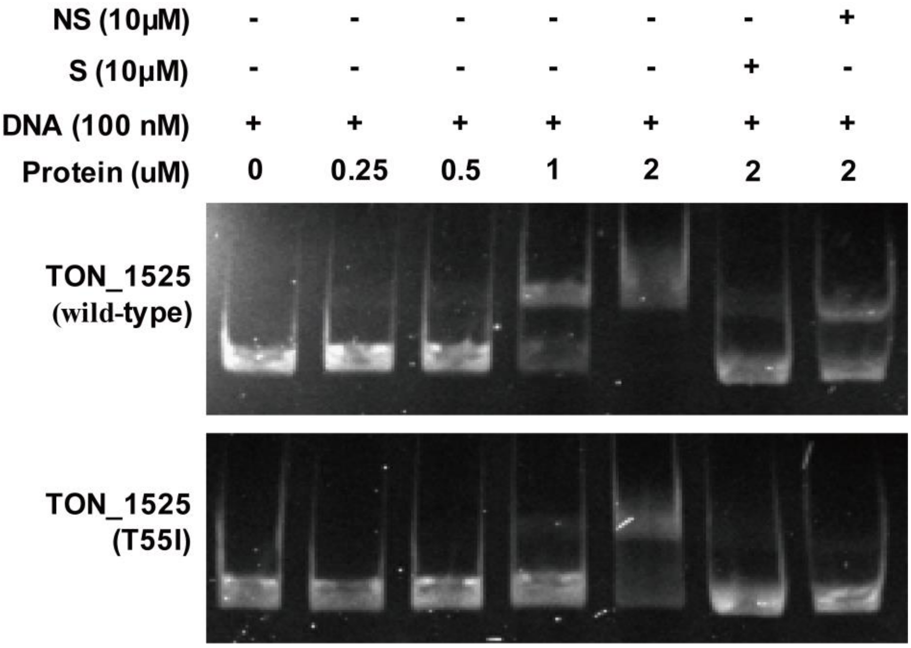

Supplementary Figure 5. Competition EMSA assay assessing the interactions between the recombinant proteins TON_1525 (wild-type) and TON_1525 (T55I) and the CODH promoter region. For competition analysis, NS and S were respectively used as nonspecific and specific DNA competitors in 100X molar excess. NS, a nonspecific cold competitor probe derived from digested pUC18 plasmid DNA; S, a specific cold competitor probe derived from the CODH promoter region.

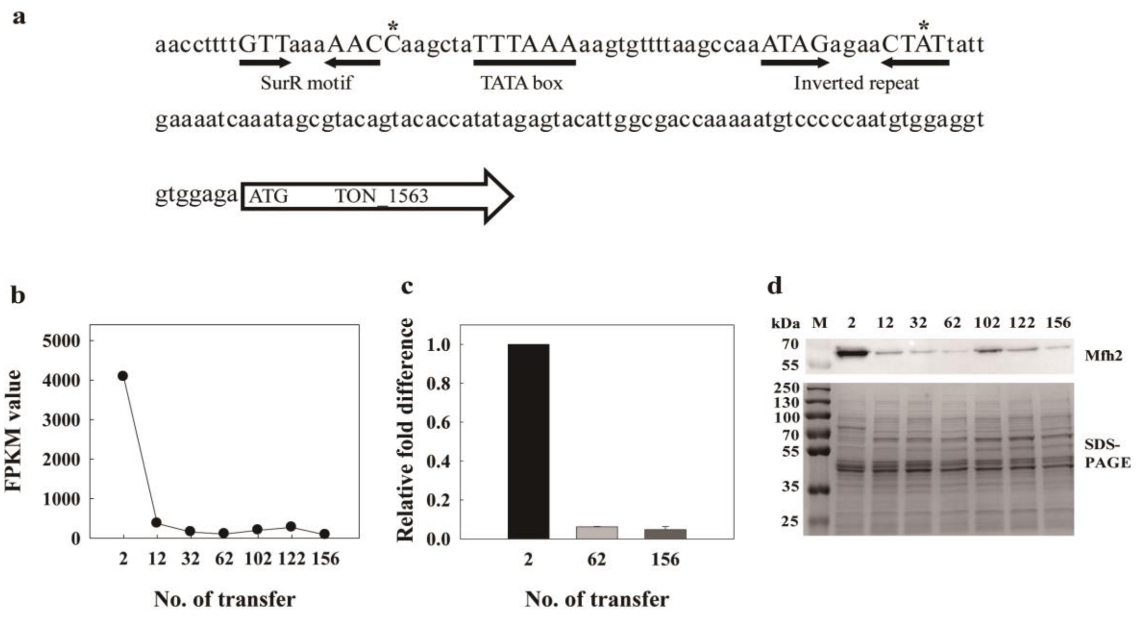

Supplementary Figure 6. Sequence analysis of the upstream region of the *mfh*2 gene cluster and the transcript level of the *mfh*2 gene during the adaptation process. (**a**) The upstream region of the *mfh*2 gene cluster, which begins with a TON_1563 gene, is displayed. The putative SurR-binding motif and a newly found inverted repeat are indicated by two converging arrows. The putative TATA box is underlined. The methylated cytosine and adenine residues that were detected only in the 156T genome are indicated by asterisks. (**b**) RNA-seq analysis of the *mfh*2 (TON_1569) gene. (**c**) RT-qPCR analysis of the mRNA abundance of *mfh*2 (TON_1569). (**d**) Western blot analysis of the Mfh2 subunit (67.2 kDa) encoded by TON_1569. The error bars indicate the standard deviations from duplicate experiments.

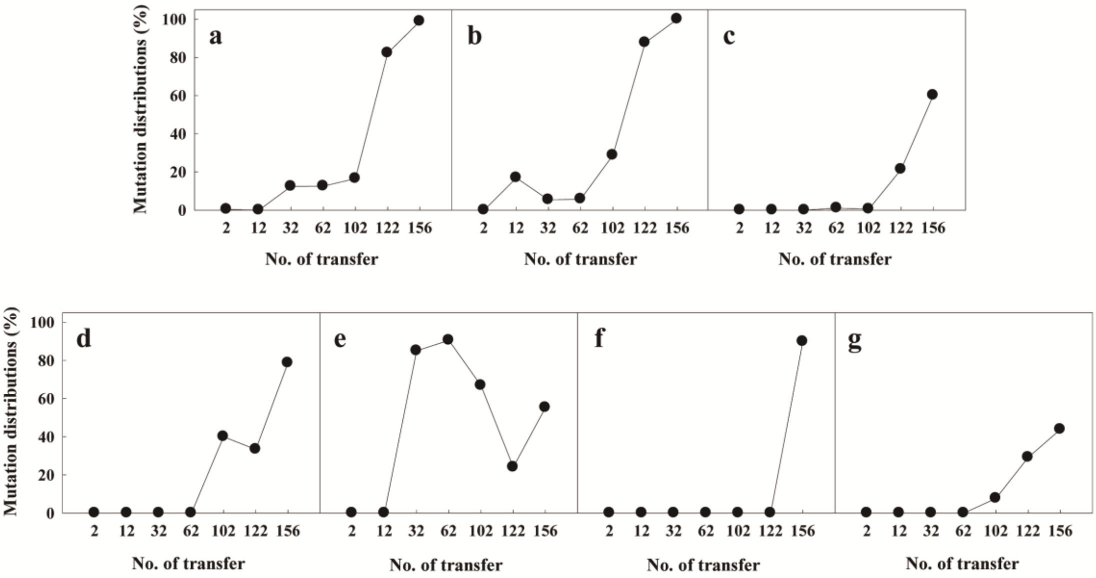

Supplementary Figure 7. The distributions of mutations in the transferred strains during the adaption process. The distribution pattern of each mutation found in the 156T genome was analyzed using RNA-seq data. The mutation distribution (%) was determined by dividing the mutation read over the total read number of each gene. The following genes were found to have mutations: TON_1525 (putative transcriptional regulator) (**a**), TON_0820 (aromatic amino acid permease) (**b**), TON_1544 (membrane protein) (**c**), TON_1548 (hypothetical protein) (**d**), TON_0982 (aminotransferase) (**e**), TON_1664 (cation transporter) (**f**) and TON_1694 (membrane-associated metalloprotease) (**g**).

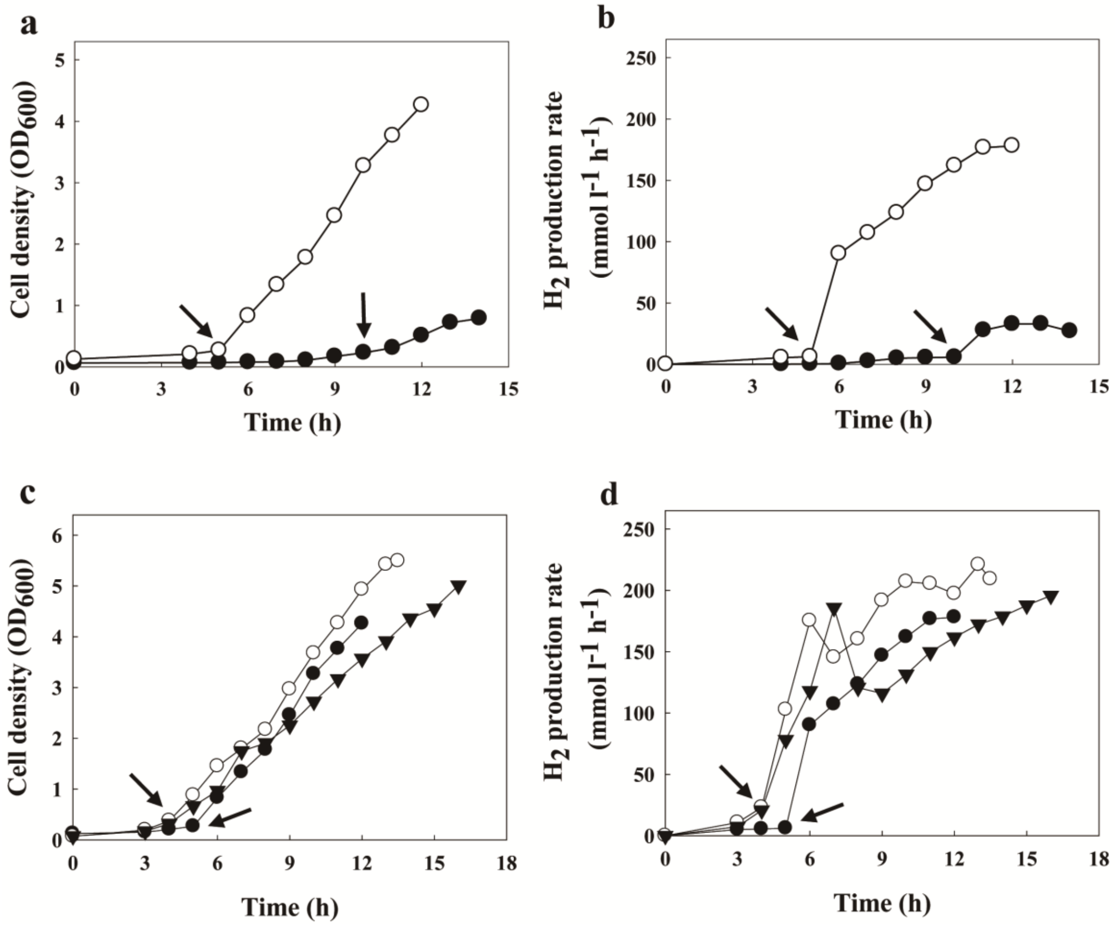

Supplementary Figure 8. Growth and H_2 production in the 156T strain at various CO flow rates. Cell density (expressed as optical density at 600 nm) (**a**) and H_2 production rate (**b**) of wild-type (closed circle) and 156T (open circle) strains were measured under a CO flow rate of 400 ml min^{-1}. Cell density (**c**) and H_2 production rate (**d**) of the 156T strain were measured under CO flow rates of 400 (closed circle), 800 (open circle) and 1000 ml min^{-1} (closed inverted triangle). The initial CO flow rate of 40 ml min^{-1} was raised when the OD_{600} reached approximately 0.3, as indicated by arrows.

Supplementary Table 1. Frequency of N^{6}-methyladenosine in the 2T and 156T strains.

Target sequences	Frequency	2T		156T		Restriction enzyme
		full	hemi	full	hemi	
GTCGA^{m6A}C	266	261	5	248	15	*Acc*I
GTATA^{m6A}C	136	125	11	116	19	*Acc*I
GTCTA^{m6A}C	661	647	2 (12)[a]	606	19 (32)[a]	*Acc*I

[a]The numbers in the parentheses represent the methylation frequencies of the motif GTAGA^{m6A}C, which is reverse and complementary to the motif GTCTA^{m6A}C.

Supplementary Table 2. H_2 production rate of the 156T strain with various feeding rates of coal-gasified syngas.

Feeding rate of coal-gasified syngas (ml min^{-1})	H_2 production rate (mmol l^{-1} h^{-1})	Specific H_2 production rate (mmol g^{-1} h^{-1})
160	108.2	182.2
320	167.0	233.2
480	198.4	254.3
640	215.4	242.0

Supplementary Table 3. Primers used in this study.

Primers	Oligonucleotide Sequences
**Construction of mutants**	
pUC118_0282del_HMG_fo_inverse_F	5'-gacctgcaggcatgcaagct-3'
pUC118_0282del_HMG_fo_inverse_R	5'-gactctagaggatccccggg-3'
TON_0820_ point mutation_F	5'-caaagaacgaggcccgggcaatactc-3'
TON_0820_ point mutation_R	5'-cccacgagtattgcccgggcctcgtt-3'
TON_1525_point mutation_F	5'-agaaagctaggaacaccatcaccatctggga-3'
TON_1525_point mutation_R	5'-tcccagatggtgatgatgttcctagctttct-3'
**Confirmation of constructs**	
TON_0820_F	5'-acagaggtgagagagatgcccgttactgatggaac-3'
TON_0820_R	5'-gaaaaaagcaaaggattacttcctgagcttgctgg-3'
TON_1525_F	5'-atgggaagtaagagcttcct-3'
TON_1525_R	5'-ctattgcttccctatctcat-3'
TON_1525_F_3’end_A	5'-tctgctcccagatggtgatga-3'
TON_1525_flanking_R	5'-ggctagacctttgtccagac-3'
**EMSA**	
Labeled_FAM_1017_150_F	5'-gagagttttactgtctctaaatgaa-3'
Unlabeled_FAM_1017_150_F	5'-gagagttttactgtctctaaatgaa-3'
1017_150_R	5'-aaccggaaaaagctggcattgttga-3'
**RT-qPCR**	
TON_1018_F	5'-gttcgagaatcctgctggtctt-3'
TON_1018_R	5'-agcaactggcaagtctgaaatg-3'
TON_1023_F	5'-tgccatcttctcggctttg-3'

TON_1023_R	5'-gctctgctatgtccattatgtattctct-3'
TON_1031_F	5'-ccgtaggaaccacgatgtacttt-3'
TON_1031_R	5'-ccgtcaaatcggcaagattaa-3'
**Confirmation of mutations in coding region**	
TON_0536_F	5'-aacgactatcttggcgttct-3'
TON_0536_R	5'-ccgagaacgtccagataatt-3'
TON_0541_F	5'-gacctcaacgagctgatgga-3'
TON_0541_R	5'-acaaggtagccagttgccgc-3'
TON_0544_F	5'-atgttgtgggagtcccagat-3'
TON_0544_R	5'-tcacttgttaagcgggtaga-3'
TON_0711_F	5'-tctgggggaactgtgacggg-3'
TON_0711_R	5'-ccacgacagggttttaatag-3'
TON_0820_F	5'-acagaggtgagagagatgcccgttactgatggaac-3'
TON_0820_R	5'-gaaaaaagcaaaggattacttcctgagcttgctgg-3'
TON_0982_F	5'-aggttctcttccacggaccg-3'
TON_0982_R	5'-tgatgcccacttcctcatgg-3'
TON_1525_F	5'-cggctaggataatcctcgac-3'
TON_1525_R	5'-cttgggataccttggctgga-3'
TON_1364_F	5'-tccatctcggccttagccgg-3'
TON_1364_R	5'-acgctccaagcaccaccttta-3'
TON_1544_F	5'-agtattctcgtacctgttgt-3'
TON_1544_R	5'-caactacatctcgcgcagga-3'
TON_1548_F	5'-ctagagtggagagcagggag -3'
TON_1548_R	5'-atgaagcaccgtaaagtgtc -3'

TON_1664_F	5'-cgcaaggaacttgtccatgg-3'
TON_1664_R	5'-caaactcaaagaagccaaaa-3'
TON_1694_F	5'-tgccggctggagaaccgggg-3'
TON_1694_R	5'-atgatcattggcatcctagc-3'
Long region deletion_TON_0536_in_F	5'-agcatgtaccagcagacgat-3'
Long region deletion_TON_0544_R	5'-tcacttgttaagcgggtaga-3'
**Confirmation of mutations in non-coding region**	
Genome position_ 1351559_F	5'-gggacgatgcccttgccgac-3'
Genome position_ 1351559_R	5'-gatcctggtgaatgccgtaa-3'

Supplementary Data, File 1 Log_2 (fold change) values of all differentially expressed genes were arranged according to their archaeal COG affiliations. Data for genes that were not assigned to any archaeal COG category were documented on the sheet named 'genes not classified'.

Supplementary Data, File 2 Comparison of methylation statuses of adenine and cytosine residues between the 2T and 156T strains. A total of 2190 and 2105 sites in both strands of the genome were detected as N^6-methyl adenine (m6A) in 2T and 156T, respectively. The sequences surrounding the modification sites were analyzed by MEME.
